# Green space availability and mental health – results from a cross-sectional study in Northwestern Germany

**DOI:** 10.1093/eurpub/ckac129.707

**Published:** 2022-10-25

**Authors:** F Sisenop, J Lindert

**Affiliations:** University of Applied Sciences Emden/Leer, Brandeis University, Emden, Germany

## Abstract

**Background:**

A relationship between green space and health has been shown in several epidemiological studies. The impact of different types of green space is still relatively unknown. To start filling this gap, we looked at associations between different green space types and health outcomes (depression and mental health).

**Methods:**

Data are obtained from a cross-sectional study (n = 479). Depression (assessed with PHQ-9) and mental health (assessed with GHQ-28) are dependent variables. Availability of green space in the surrounding neighborhood was assessed as independent variable by the percentage of green space ( > =1ha) within a 250m radius participants residence. Survey data were analyzed using IBM SPSS 26 and Geo data using QGIS 3.18.0.

**Results:**

N = 479 participants of a cross-sectional study in 2018 provided data (49.4%, n = 240 women; 49.6%, n = 239 men). Participants had a mean age of 57.55 years (SD: 18.80, min-max:18-95), majority (75.2%, n = 360) were married or partnered, had a lower educational qualification than A-levels equivalent (56.8%, n = 272), were not employed (53%, n = 254), had a net household income of at least 3. 000€ per month (40.1%, n = 192) and at least sometimes financial worries (51.4%, n = 246). Green areas without agricultural areas show an association with frequency of depression (B(SE)=0.056(0.024), p = 0.018). This contrasts with green spaces including agricultural areas, where there is no statistically significant association (B(SE)=0.007(0.012), p = 0.564).

**Discussion:**

We found an association between type of green space and depression. Further studies are needed to establish a grid for assessing characteristics and quality criteria of green spaces. However, it can already be assumed that there is an association between quality of green spaces and psychosocial outcomes.

